# A functionally integrated cross-tissue alternative splicing program during short-term calorie restriction

**DOI:** 10.1093/lifemeta/loaf046

**Published:** 2026-02-12

**Authors:** Daniel P Phillips, Sharon E Mitchell, Davina Derous, John R Speakman

**Affiliations:** School of Biological Sciences, University of Aberdeen, Aberdeen AB24 2TZ, Scotland, United Kingdom; School of Biological Sciences, University of Aberdeen, Aberdeen AB24 2TZ, Scotland, United Kingdom; School of Biological Sciences, University of Aberdeen, Aberdeen AB24 2TZ, Scotland, United Kingdom; School of Biological Sciences, University of Aberdeen, Aberdeen AB24 2TZ, Scotland, United Kingdom; Shenzhen Key Laboratory of Metabolic Health, Center for Energy Metabolism and Reproduction, Shenzhen Institute of Advanced Technology, Chinese Academy of Sciences and Shenzhen University of Advanced Technology, Shenzhen, Guangdong 518055, China; State Key Laboratory of Molecular Developmental Biology, Institute of Genetics and Developmental Biology, Chinese Academy of Sciences, Beijing 100101, China; Institute of Health Sciences, China Medical University, Shenyang, Liaoning 110122, China

**Keywords:** calorie restriction, alternative splicing, aging, RNA

## Abstract

Alternative splicing (AS) involves production of several different RNA molecules from a single pre-mRNA. Calorie restriction (CR) is a sustained calorie deficit without malnutrition, which extends health and lifespan. AS is dysregulated in aging but less so following CR, suggesting a role for AS in the beneficial effects of CR. To test the hypothesis that AS is involved in the CR response and explore its tissue specificity, male C57BL/6 mice were exposed to 3 months of graded CR (0–40% at 10% increments) and RNA sequencing data from six tissues (epididymal white adipose tissue [eWAT], liver, hypothalamus, gastrocnemius muscle, testes, and stomach) were analyzed to provide a multi-tissue characterization of differential AS (DAS). The number of differentially expressed splicing regulators increased with CR levels in all tissues but primarily the muscle, eWAT, and liver. The total number of DAS genes also increased with increasing CR levels and was largely tissue-specific. Most DAS genes were not differentially expressed. DAS was functionally integrated across tissues with the same processes being overrepresented: namely, mitochondria and oxidative phosphorylation, transcription and translation, and quality checking and degradation of RNA and especially proteins. This study demonstrates that short-term CR evokes a functionally integrated cross-tissue AS response in mice that is largely independent of expression changes.

## Introduction

Age is a major risk factor for most chronic diseases including type 2 diabetes mellitus [1], neuro-degenerative diseases, such as ­Alzheimer’s and Parkinson’s disease [2], cardiovascular disease [3], and cancer [4]. Geroscience is concerned with elucidating the mechanisms that underpin senescent phenotypes [5]. These ­“Hallmarks of Ageing” relate to homeostatic and developmental mechanisms at the genomic (e.g. genetic instability and degradation of telomeres), epigenomic (e.g., differential chemical modifications to the nucleosome and RNAs), and proteomic (e.g. altered proteostasis via inefficient protein assembly, disassembly, and localization) levels [6]. More recently, dysregulation of transcription and post-transcriptional processes, particularly alternative splicing (AS), has emerged as novel, widely conserved, and potentially causal factors in the aging process [7].

AS involves the production of multiple different RNA molecules from a single pre-mRNA. It is catalyzed by a large (approximately 3 MDa) multi-subunit ribonucleoprotein complex called the “spliceosome” and regulated by the combinatorial binding of pre-mRNA and the spliceosome by cis- and trans-acting splicing factors [8]. In addition, various auxiliary RNA-binding proteins and non-coding RNAs regulate the splicing machinery [9]. In recent years, dysregulation of AS has been associated with aging across several taxa [10–17].

Despite improved understanding of the molecular hallmarks of aging, there has been limited pharmaceutical efficacy in slowing the rate of aging in humans [18]. In contrast, calorie restriction (CR) is recognized as an effective non-genetic intervention to retard the rate of aging [19, 20]. The benefits of CR are greater: (i) the earlier it is initiated; (ii) the longer it is maintained; and (iii) the greater the level of restriction, up to at least 65% restriction. Although the precise mechanism(s) responsible for the benefits of CR remain elusive, it targets many of the hallmarks of aging including AS. For example, Tabrez *et al.* found hundreds of differentially regulated AS events during CR in adult hermaphrodite *C. elegans* and thousands in female Swiss Webster x DBA/C57BL6 mice under 30% CR [21]. The number of AS genes increases as the duration of CR increases in the mice. Heintz *et al.* also suggested that AS drives lifespan extension during CR in *C. elegans* by showing that age is associated with increased intron retention and unannotated splice junctions, indicative of greater splicing noise, which were reversed by CR [15]. Rhesus monkeys exposed to 30% CR for 2 years had 455 exons differentially included in RNAs of 387 genes in the liver [22]. Of those AS loci, less than 6% were also differentially expressed.

Although previous studies have improved our understanding of how AS is linked to aging and CR, the focus has been on single tissues at single CR levels, which leaves open the question of cross-tissue coordination of the AS response. In this study, we analyzed existing RNA sequencing (RNA-seq) datasets from four tissues {epidydimal white adipose tissue (eWAT) [23], liver [24], hypotha­lamus [25], and stomach [26]} as well as novel datasets from a further two tissues (gastrocnemius muscle and testes) from the same individual male C57BL/6 mice following 3 months at different levels of CR (between 0 and 40%) [27] to assess system-wide changes in AS. We discovered that CR led to a significant AS response that was broadly proportional to the level of CR, mostly independent of differential expression (DE), and largely tissue-specific. These results are consistent with the hypothesis that aging causes disruptions in the splicing process which are abrogated by CR.

## Results

### Gene expression response to graded CR

**Figure 1:**
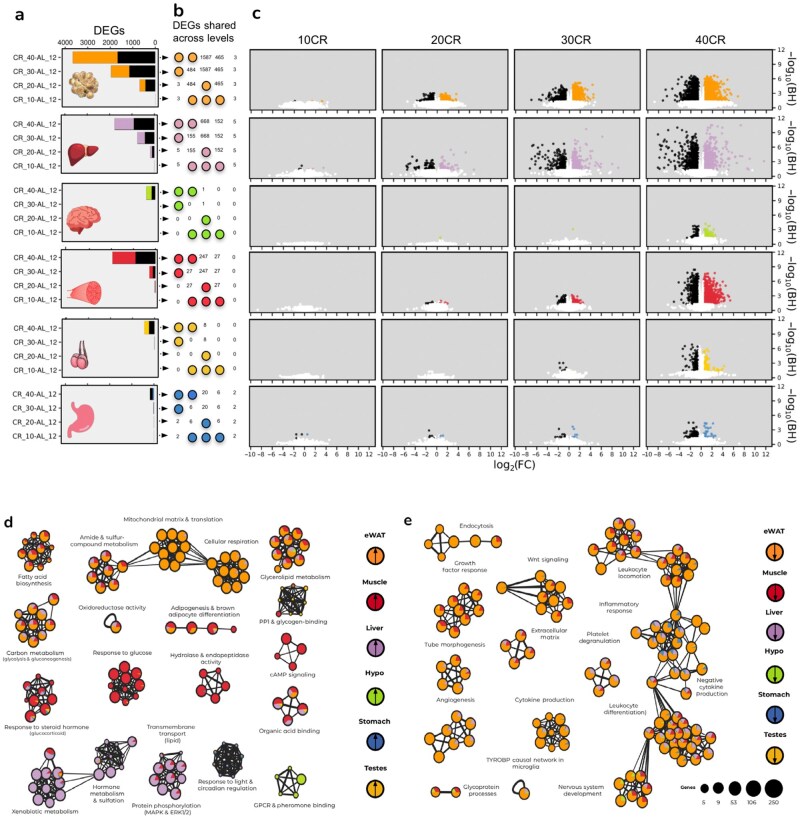
Differential gene expression analysis of male C57BL/6 mice exposed to graded levels (10%−40%) of short-term (3-month) CR compared to mice fed a control diet of AL feeding for 12 h (AL12) each day. (a) Bar plots showing the number of DEGs at each CR level compared to AL12 in each of the six tested tissues. DEGs of the eWAT, liver, hypothalamus, gastrocnemius muscle, testes, and stomach are shown. Colored bar represents up-regulated gene number following CR and black bar represents down-regulated gene number following CR. (b) Combination matrix of DEGs common between CR levels within each tissue irrespective of direction. Numbers indicate the number of common DEGs and their position indicates the CR levels which share that number of DEGs. Colored points indicate the other CR conditions which are being ignored. (c) Aligned volcano plots showing DE results (log_2_(FC) in expression and adjusted *P* value) for each tissue at each CR level. (d) Enrichment map showing MetaScape functional enrichment results of up-regulated DEGs from each tissue combining all CR levels. (e) Enrichment map showing MetaScape functional enrichment results of down-regulated DEGs from each tissue combining all CR levels. Nodes are scaled by the maximum gene count per functional category and subset by counts per tissue. Edges represent genes shared by different functional nodes.

We first analyzed transcriptome-wide expression responses to graded levels of CR (ie, 10% CR [10CR], 20CR, 30CR, and 40CR compared to 12-h *ad libitum* feeding [12AL]) to provide regulatory context for the differential AS (DAS) analysis in the same mice. The number of differentially expressed genes (DEGs; absolute log_2_(fold change) [log_2_(FC)] > 0.5 and false discovery rate (FDR) < 0.05) compared to 12AL increased with the levels of CR in all tissues, except the testes and hypothalamus. In these two tissues, significant changes in gene expression compared to 12AL were only observed at the highest CR levels (Fig. 1a). Combination matrices indicating overlap of DEGs between CR levels (compared to 12AL) in each tissue are shown in Fig. 1b.

The eWAT had the most DEGs relative to 12AL (4126 unique DEGs across 10CR–40CR), followed by muscle (1982 unique DEGs) and liver (1,883 unique DEGs). The testes (464 unique DEGs), hypothalamus (399 unique DEGs), and stomach (184 unique DEGs) exhibited comparatively weaker expression responses to CR (Fig. 1a). Despite the eWAT featuring the most DEGs, the liver had the most substantial changes in gene expression (relative to 12AL) in terms of statistical significance and effect sizes (Fig. 1c).

DEGs responding to CR were enriched (FDR < 0.05) as targets for numerous transcription factors. Most notable was the peroxisome proliferator-activated receptor-alpha (PPAR-α), a transcription factor coordinating lipid oxidation. Targets of PPAR-α were significantly enriched among genes up-regulated in the muscle, testes, liver, and eWAT (Supplementary Fig. S1). Targets of the lipid uptake and adipogenesis transcription factor PPAR-γ were similarly up-regulated in these tissues but not in the liver, whereas targets of transcription factors controlling cholesterol and fatty acid synthesis, sterol regulatory element binding transcription factor 1 (Srebf1) and MLX interacting protein-like (MLXIPL or carbohydrate-responsive element-binding protein [ChREBP]), were enriched among DEGs up-regulated in the eWAT, testes, and muscle but down-regulated in the liver (Supplementary Fig. S1). There was also a substantial enrichment for target genes of growth factors and inflammation-related transcription factors being down-regulated in the eWAT, particularly those of the Jun (Jun and Junb), Erg, and SMAD (Smad1, Smad3, and Smad7) families (Supplementary Fig. S1). Other notable transcription factor signatures included enrichment of targets of steroidogenesis and sex determination transcription factors (Nr5a1, Nr0b1, and Sf1) among genes down-regulated in the testes, and enrichment of targets of circadian rhythm and chromatin modifying transcription factors among genes up-regulated in the stomach (and also weakly in the liver) but down-regulated in the muscle (Supplementary Fig. S1).

Significantly enriched functions of DEGs were broadly tissue-specific and consistent with the transcription factor enrichment results (Fig. 1d and e). Lipid biosynthesis and metabolism-related genes were overrepresented among up-regulated genes in the eWAT, muscle, and liver, with some enrichment also in the testes and hypothalamus. Oxidoreductase activity and metabolism of carbon (e.g. glycolysis and gluconeogenesis) and amide and sulphur-containing compounds showed a similar pattern of enrichment following CR (Fig. 1d). The eWAT also exhibited a strong overrepresentation for increased expression of genes involved in mitochondrial components and translation, as well as oxidative phosphorylation (OXPHOS), which was partially shared with the liver (Fig. 1d). Conversely, genes up-regulated in the liver were overrepresented for functions related to xenobiotic metabolism, which was weakly shared with the muscle, eWAT, and stomach, as well as sulfation of hormones, which was almost uniquely enriched in the liver (Fig. 1d). Up-regulated genes in the hypothalamus were uniquely enriched for G-protein coupled receptor (GPCR) and pheromone-binding functions, and circadian regulation was enriched in genes up-regulated primarily in the stomach (Fig. 1d). Functional enrichment of genes down-regulated by CR was largely similar across tissues but dominated by the eWAT, and was related to immune functions, angiogenesis, and growth factor signaling (Fig. 1e).

**Figure 2:**
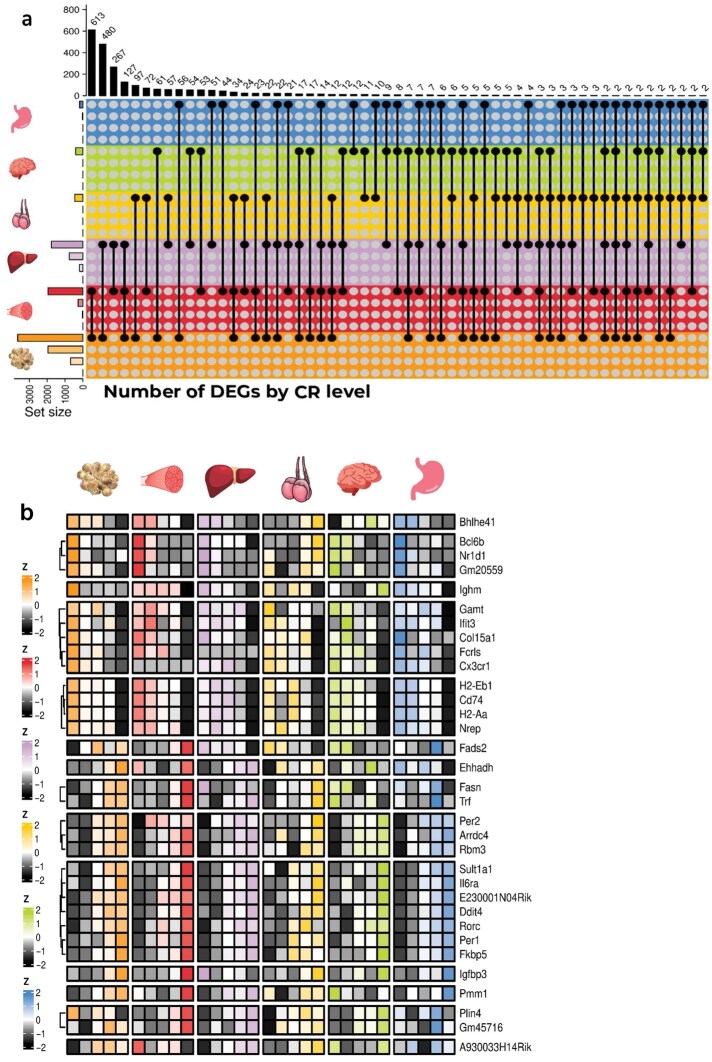
Overlap of DEGs between six tissues of male C57BL/6 mice exposed to graded levels (10%−40%) of short-term (3-month) CR, compared to mice fed a control diet of AL feeding for 12 h (AL12) each day. (a) Upset plot showing the number of DEGs common between 40CR and AL12 across each of the six tissues, regardless of their direction of change in each tissue. (b) Combined heatmap showing the scaled (by gene, within-tissue) log_2_(CPM) expression of 33 DEGs common across at least four tissues following short-term CR (at 40CR compared to AL12).

Given that the strongest DE response (compared to 12AL) was consistently at 40CR in each tissue, focus was made on the intersections of DEGs between tissues only at this level. The most substantial cross-tissue regulation of gene expression at 40CR was observed between the eWAT and the muscle, which shared 613 DEGs, followed by the eWAT and the liver (*n *= 480), the muscle and the liver (*n *= 267), and the eWAT, the muscle, and the liver (*n *= 127; Fig. 2a). Overall, there were 1,373 DEGs common between any two tissues, 216 common between any three tissues, 33 between any four tissues (Fig. 2b), and 6 between any five tissues. Only two genes, *H2-Aa* and *H2-Eb1*, were differentially expressed following CR in all six tissues (FDR < 0.05 and log_2_(FC) > 0.05) with decreased expression in all tissues (Fig. 2b). Expression profiles of these two genes as well as a selection of the 33 DEGs shared by at least four tissues with recognized functions most relevant to CR physiology are shown in Supplementary Fig. S2.

### DE of splicing regulators

**Figure 3:**
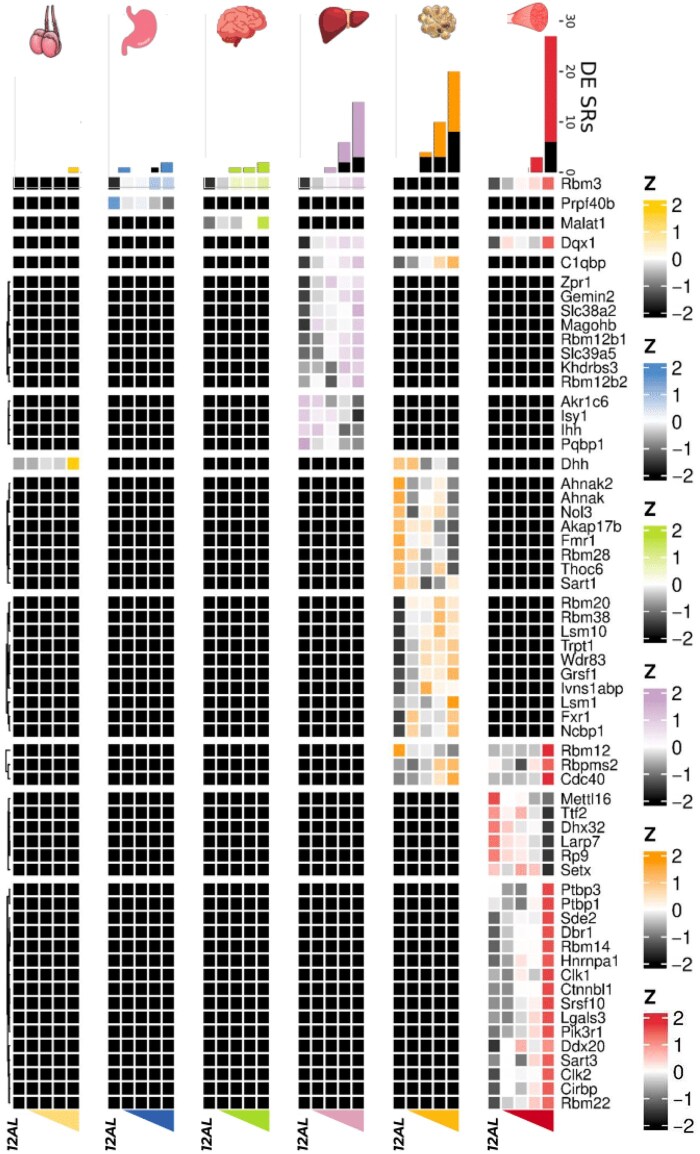
Overlap of differentially expressed SRs between six tissues of male C57BL/6 mice exposed to graded levels (10%−40%) of short-term (3-month) CR, compared to mice fed a control diet of AL feeding for 12 h (AL12) each day. Combined bar plots and heatmap show both the number of differentially expressed SRs at each CR level (compared to AL12) within each of the six tissues (testes, stomach, hypothalamus, liver, eWAT, and gastrocnemius muscle) and their direction of change in expression (black indicates down-regulation), and the log_2_(CPM) expression profiles of differentially expressed SRs common across at least two tissues (at 40CR), scaled by gene within each tissue.

The number of differentially expressed splicing regulators (SRs) compared to 12AL increased as the levels of CR increased in all tissues (Fig. 3). SRs were generally upregulated following CR, ­especially in the muscle, which had the most (*n *= 27) overall (Fig. 3). The eWAT had 23 differentially expressed SRs followed by the liver (*n *= 15), with far fewer in the stomach (*n *= 2), hypothalamus (*n *= 2), and testes (*n *= 1; Fig. 3). Most differentially expressed SRs were tissue-specific, but seven were differentially expressed in more than one tissue (at 40CR compared to 12AL; Fig. 3), including the cold-sensitive SR Rbm3, which was up-regulated in the muscle, liver, hypothalamus, and stomach (Supplementary Fig. S3). Other SRs differentially expressed (at 40CR relative to 12AL) across multiple tissues were Dqx1 (up-regulated in the muscle and the liver), mitochondrially localized C1qbp (p32; up-regulated in the eWAT and the liver), Dhh (up-regulated in the testes and down-regulated in the eWAT), Rbm12 (up-regulated in the muscle and down-regulated in the eWAT), Rbpms2, and 6Cdc40 (both up-regulated in the muscle and the eWAT; Supplementary Fig. S3).

### AS response to graded CR

**Figure 4:**
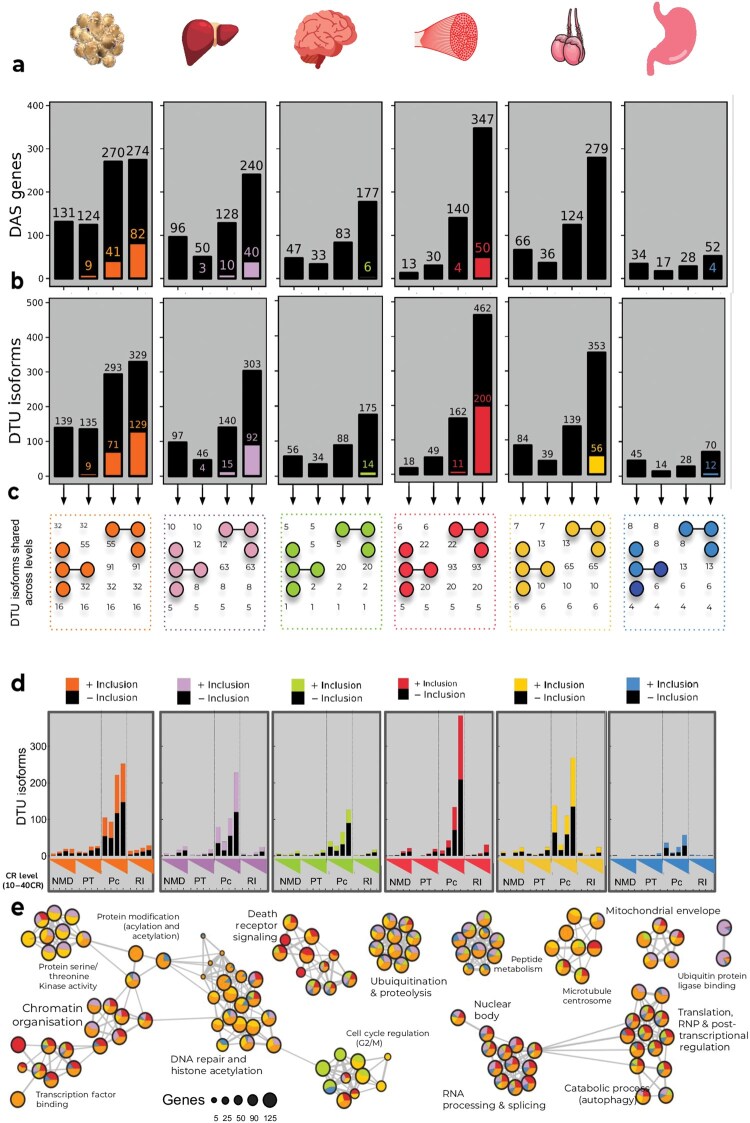
DAS and DTU analyses of male C57BL/6 mice exposed to graded levels (10%−40%) of short-term (3-month) CR, compared to mice fed a control diet of AL feeding for 12 h (AL12) each day. Colors coding for all plots are shown: orange, eWAT; purple, liver; green, hypothalamus; red, gastrocnemiusmuscle; yellow, testes; blue, stomach. (a and b) Bar plots showing the number of DAS genes (a) and DTU isoforms (b) at each CR level compared to AL12 in each of the six tested tissues, with the number of respective genes or isoforms that are differentially expressed (denoted by their tissue-specific color). (c) Combination matrix of DTUs common between CR levels (compared to AL12) within each tissue. Numbers in the figure represent the numbers of common DTUs and their positions indicate the CR levels which share that number of DTUs. Colored points indicate the other CR conditions which are ignored. (d) Bar plots showing the number of DTU isoforms at each CR level compared to AL12 in each of the six tested tissues, stacked according to their annotated isoform biotypes (NMD, nonsense mediated decay-prone; PT, processed transcript; Pc, protein codin; RI, retained intron) at each CR level (compared to AL12) within each tissue, along with their direction of change in inclusion (PSI). Black indicates a reduction in PSI. (e) Enrichment map showing MetaScape functional enrichment results of DTU gene loci from each tissue combining all CR levels. Nodes are scaled by gene maximum gene count per functional category and subset by counts per tissue. Edges represent genes shared by different functional nodes.

The hypothalamus, eWAT, and testes tended to have more expressed isoforms per gene than the muscle, stomach, and liver ­(Supplementary Fig. S4). Similar to DE, the number of both DAS genes and differential transcript usage (DTU; compared to 12AL) increased with increasing CR levels (Fig. 4a and b). A notable exception was the eWAT, which instead showed a significant increase in the number of DAS genes from 10CR–20CR to 30CR–40CR (Fig. 4a). Given their isoform-level resolution, a focus was made on DTU. Unlike DE, the muscle had the widest differential transcript response to CR at 40CR (compared to 12AL), with 462 isoforms exhibiting DTU (and 560 unique isoforms across all levels), followed by the testes (353 at 40CR, 494 overall), the eWAT (329 at 40CR, 676 overall), the liver (303 at 40CR, 468 overall), the hypothalamus (175 at 40CR, 310 overall), and the stomach (70 at 40CR, 117 overall; Fig. 4b).

While there was a general increase in DAS with increasing CR levels, the trend seen for DEGs at lower CR levels being subsets of those at higher levels was much weaker for DAS and DTU (Fig. 4c). DAS patterns consistent across CR levels were therefore taken as more reliable features of the AS response. In the eWAT, 32 isoforms exhibited differential isoform usage (compared to 12AL) across 20CR–40CR (and 16 across 10CR–40CR), including some loci involved in lipid metabolism (*Copg1-203* and *Agpat1-202*), the TCA cycle (*Idh3b-204*), OXPHOS (*Ndufab1-208/205* and *Uqcr10-201/203*), constituents of the ribosome (*Mrps35-202*, *Rpl28-203*, and *Rplp2-201/203*), epigenetic regulation (*Mbd6-201*), apoptosis and autophagy (*Siva1-201/204* and *P4hb-202*), and a lncRNA host gene involved in cancer progression (*Snhg6-203*; Supplementary Fig. S5a). Eight isoforms exhibited differential isoform usage relative to 12AL across 20CR–40CR in the liver (and five across 10 CR–40CR). These were immune system-related gene *Klhl5-202*, global SR *Rbm39-203*, oxidative-stress mediated signaling and autophagy-associated oncogene *Txndc9-202*, p53-regulated snoRNP-assembly and cellular senescence gene *Nolc1-202*, detoxification gene *Gstt2-201*, transcriptional regulator *Nolc1-202*, developmental gene *Klhl5-202*, and a relatively unknown transcription factor *Zfp865-202* (Supplementary Fig. S5b). Only two isoforms featured differential isoform usage compared to 12AL across 20CR–40CR in the hypothalamus. These were RNA processing GTPase *Hbs1l-201* and guanine nucleotide binding protein (of GPCRs) *Gnas-218*, with only the former exhibiting differential isoform usage across all four CR levels ­(Supplementary Fig. S5c). In the muscle, 20 isoforms featured DTU relative to 12AL across 20CR–40CR, including regulator of transcription and AS *Ddx5-201*, cholesterol biosynthesis enzyme *Sc5d-201/202*, *Ociad1-202*, which is involved in stem cell homeostasis and rejuvenation, spliceosome component *Aar2-201/202*, and the transmembrane protein *Tmem69-201/202*, with five DTU isoforms across all levels ­(Supplementary Fig. S5d). In the testes, there were 10 isoforms with differential isoform usage across 20CR–40CR, including *Crisp1-201/207*, which is important to sperm development and function, ribosomal protein 7 (*Rpl7-201/205*), cell cycle regulator *Arpp19-210*, ­endoplasmic reticulum membrane protein *Retreg2-202*, and the transforming growth factor-beta (TGF-β) target gene *Tsc22d1-214*, with six DTU isoforms across all CR levels ­(Supplementary Fig. S5e). Only six isoforms had differential isoform usage across 20CR–40CR in the stomach, namely two isoforms from TGF-β target and mitochondrial homeostasis gene *Vps39-201/202*, interferon regulatory factor *Irf3-202*, redox regulator and glucose receptor recycler *Glrx2-202*, transmembrane protein *Ginm1-205*, and SR *Hnrnpa3-204*, and four DTU isoforms shared across all levels (Supplementary Fig. S5f).

Most (94%) gene loci with differential usage isoform (and DAS) were not also differentially expressed, indicating that AS is an independently regulated response to CR (Fig. 4a and b). Nonetheless, the number of co-regulated (differentially expressed and differentially alternatively spliced or DTU) loci increased with CR level and was often more than predicted by chance (hypergeometric tests, *P *< 0.001; Fig. 4a and b), indicating overlapping regulation of the transcription and AS of these transcripts. The number of all DTU isoform biotypes (relative to 12AL) increased broadly in line with CR level. Most DTU isoforms were protein coding in all tested tissues (Fig. 4d), suggesting that the AS response to CR largely functions to regulate and remodel proteome composition, with a weak tendency for reduced inclusion. The majority of protein-coding isoforms were accompanied by approximately equal numbers of isoforms annotated as predicted for nonsense mediated decay, processed transcripts, and retained intron transcripts (Fig. 4d). A tiny fraction of isoforms with other biotypes were excluded for visualization (Supplementary Fig. S6).

Genes of DTU isoforms were input into MetaScape to identify enriched functional signatures of CR-related AS in each tissue. All six tissues exhibited an enrichment for ribosome and translation, stress response, protein ubiquitination and degradation, post-transcriptional regulation of gene expression (including via RNA stability and splicing), centromere, transcription factors, protein localization, death receptor signaling, and DNA binding among DTU genes (Fig. 4e), albeit with an overall tendency for stronger enrichment in the eWAT, muscle, and liver than other tissues. There was an additional and notable enrichment of DTU among cell cycle-related genes in the hypotha­lamus and, to some extent, the testes (Fig. 4e).

**Figure 5:**
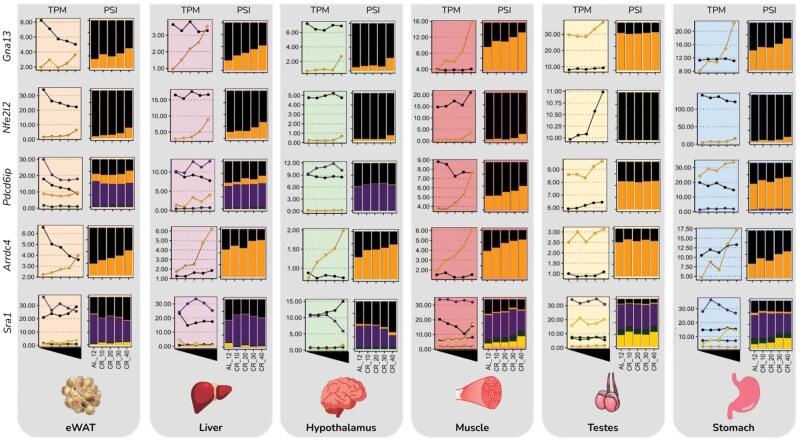
Overlap of selected DTU isoforms between six tissues of male C57BL/6 mice exposed to graded levels (10%−40%) of short-term (3-month) CR, compared to mice fed a control diet of AL feeding for 12 h (AL12) each day. Plots showing the isoform expression (indicated as TPM reads) and AS profiles (isoform inclusion, measured as percentage splice-in; PSI) of a selection of genes with DTU (compared to AL12) common across at least two tissues following CR.

The specific AS features common across tissues (at 40CR compared to 12AL) were explored to provide AS-level resolution to the previous cross-tissue functional theme comparison of the mouse CR response (Supplementary Fig. S7a). The greatest number of cross-tissue DTU isoforms was found between the eWAT and the muscle (*n *= 20), followed by the muscle and the liver (*n *= 14), the eWAT and the liver (*n *= 13), the muscle and the hypothalamus (*n *= 11), and the muscle and the stomach (*n *= 11), with less than 10 DTU isoforms common between all other combinations (Supplementary Fig. S7a). There were 65 unique isoforms exhibiting DTU at 40CR relative to 12AL across any two tissues (Supplementary Fig. S7a and b), 11 across any three tissues, 6 between any four tissues, 2 between any five tissues, and none between all six tissues (Supplementary Fig. S7a and b). AS profiles of a selection of these cross-tissue DTU isoforms are shown in Fig. 5, namely G protein subunit alpha 13 (*Gna13-201/202*), arrestin domain-containing protein 4 (*Arrdc4-201/202*), programmed cell death 6 interacting protein (*Pdcd6ip-201/202*), NFE2 like BZIP transcription factor 2 (*Nfe2l2-201/202*), and steroid receptor RNA activator 1 (*Sra1-206*; Fig. 5a).

Overall, these results characterize tissue-specific DAS responses to short-term CR that were proportional to CR levels and independently regulated from DE. While the specific AS loci were largely tissue-dependent, many functions including apoptosis, splicing, epigenetic regulation, ribosome and translation, and RNA and protein quality control were often shared across tissues.

## Discussion

Aging is the strongest predictor of disease-onset and death. In contrast with limited pharmaceutical success at slowing the human aging process, approaches such as CR are gaining increased attention. Numerous pro-longevity mechanisms linked to CR have been identified that translate to improved health span and survival in non-human primates [28] as well as improved markers of health in human trials [29]. Recently, DAS has been associated with the aging process in both animals and humans, and the association was complemented by evidence suggesting that CR leads to improved health span and longevity partly by delaying the onset of these changes [30]. However, two fundamental questions regarding the role of AS during CR remain to be answered. These are (i) whether the AS response to CR is similar across different tissues and (ii) whether the AS response is proportional to the level of CR, which would implicate it in the dose-dependent improvement of health by CR in animal models. By analyzing RNA-seq data from six tissues (eWAT, liver, hypothalamus, muscle, testes, and stomach) in male C57BL/6 mice undergoing increasing levels of CR (10%–40%) for 3 months, we uncovered transcriptome-wide changes in AS across both CR levels and tissues, and compared them to concurrent changes in gene expression.

We identified widespread differential gene expression in response to CR including both tissue-specific and non-specific patterns. Only two genes, *H2-Aa* and *H2-Eb1*, were differentially expressed across all six tested tissues. These two major histocompatibility complex (MHC) II genes, which play an important role in initiating inflammation via antigen presentation and are known to increase with aging [31], were down-regulated across tissues following CR (Fig. 2b), as previously reported [32]. This suggests that the benefits of CR involve counteracting age-associated transcriptional changes. Similar to previous studies, we discovered that CR increased DE of AS regulators, largely upregulating them in the muscle, eWAT, and liver. The number of differentially expressed SRs was proportional to the level of restriction, consistent with a role for AS in mediating the effects of CR on health and longevity. Furthermore, specific differentially expressed SRs were largely tissue-specific, suggestive of their down-stream tissue-specific regulation of AS. However, a small number of SRs exhibited an expression response to CR shared across multiple tissues, suggesting the regulation of a cross-tissue AS program. One such case was Rbm3 (Fig. 3a), which significantly increased in expression following CR in four of the six tissues (the muscle, liver, hypothalamus, and stomach). Rbm3 is an RNA binding protein known to regulate and be regulated by AS, and consistently exerts physiologically protective effects by mechanisms including regulation of translation and the cell cycle [33]. CR is associated with a reduction in core body temperature which has been directly implicated in its health benefits [34, 35]. Interestingly, Rbm3 expression is strongly induced by cold temperatures and has a causative role in the neuroprotective effects of medically induced hypothermia [36, 37], possibly suggesting that Rbm3 plays a similarly direct role in the protective effect of CR and that such outcomes are potentially mediated by regulating AS.

CR led to DAS, which was also observed in previous work [15, 21, 22], although this response was smaller than DE (hundreds of loci versus thousands). Consistent with their greater DE of SRs (and overall DE), the muscle, eWAT, and liver were the tissues with the most extensive AS changes. Interestingly, the testes also had a pronounced AS response to CR (the second most DAS loci overall), despite having a comparatively lower DE response that included only a single SR. This may suggest an outsized role for AS in responding to CR in the testes [38]. The number of loci featuring DAS following CR (compared to 12AL) broadly increased in line with the level of restriction, thus implicating changes in AS as a potentially meaningful factor in the dose-dependent improvement of health and lifespan by CR. There was, however, less overlap of DAS loci across CR levels than observed for DEGs, consistent with greater stochasticity in the regulation of AS compared to gene expression [39, 40]. Similar to previous studies [22], AS was an independent response to CR, with only a small fraction of DAS loci also being differentially expressed in any given tissue. However, the overlap of differentially expressed and DAS genes was greater than predicted by chance, potentially indicating the co-regulation of transcription and post-transcriptional processes. However, this may also reflect the inability to precisely distinguish AS from isoform-specific transcriptional regulation using the current method.

Another outstanding question is whether AS during CR promotes more efficient RNA surveillance and nonsense-mediated decay [21, 39] or regulates transcript isoform translation [18]. In this study, the majority of isoforms exhibiting DTU were protein coding, suggesting that regulation of proteome composition might be the primary outcome of the AS response to CR. Only a small fraction of DTU was related to intron retention, which has been suggested as a distinctive marker for aging and therefore might be expected to be enriched among AS loci following CR. However, although the inclusion of processed transcripts and retained-intron isoforms did tend to reduce with CR level in the eWAT, inclusion of these three “junk” biotypes was mostly balanced between increase and reduction in other tissues, and often increased inclusion at one or more CR levels, suggesting that each may have a more complex role in physiological adaptation to CR than simply being the reversal of AS “errors.”

Previous studies have identified hundreds of AS loci following CR in given tissues. Here we showed that the majority of these loci are not shared across tissues. However, in contrast to the specific loci that were differentially spliced, the functions enriched among these loci within each tissue were broadly similar and included a number of housekeeping and hallmark dysfunctions of aging. For instance, affected processes included catabolic processes such as autophagy and cell death, DNA and transcription factor binding, and particularly RNA and protein turnover (i.e. protein ubiquitination). Interestingly, loci known to be involved in RNA binding and the regulation of AS were noticeably enriched among DAS loci following CR. This is consistent with the hypothesis that a feedforward dysregulation of the splicing machinery is important in the aging process and may be a primary mechanistic target of CR. Components of the mitochondrial matrix were also generally enriched among DAS loci, suggesting AS as one means of metabolic reprogramming during CR, and thus providing evidence for the targeting of a supposed energy-splicing resilience axis hypothesized to drive aging [41, 42].

A small subset of DAS loci were common across multiple tissues, and may reflect more important roles in the benefits of CR. One of these was guanine nucleotide binding protein alpha 13 (*Gna13*) encoding Gα13, which is involved in many biological processes by mediating signal transduction of GPCRs. Gα13 is implicated in both positive and negative regulation of tumorigenesis [43], and increases in expression with age in 5XFAD mice [44]. Moreover, it is a biomarker for β-amyloid (Aβ) deposition and phosphorylated tau (p-tau) in Alzheimer’s disease (AD) in humans [44]. Interestingly, one of the primary mechanisms by which Gα13 might be involved in AD progression is through disruption of AS [45]. In mice, there are two isoforms of *Gna13* annotated in GRCm38. The canonical isoform (201) is 6160 bp with four exons encoding 377 amino acid residues, while the alternative (202) isoform is just 914 bp with only two exons encoding a 173-amino acid protein with severe disruption to the overall Ga domain including missing G2-5 motif regions. Gna13-202 showed increased inclusion (at 40CR compared to 12AL) in all tissues except for the testes, concurrent with reduced inclusion of Gna13-201 (Fig. 5), suggesting reduced Gα functionality with presently unknown but likely system-wide consequences. Another gene featuring cross-tissue AS in response to short-term CR was nuclear factor erythroid 2-related factor 2 (Nfe2l2), which is a transcription factor regulating a range of target genes with protective functions, including antioxidation and metabolic plasticity [46, 47]. Nfe2l2 has reduced expression with age, leading to the induction of senescence and various pathological age-associated phenotypes [48]. Similar to Gna13, Nfe2l2 has two annotated isoforms in mice. The canonical isoform (201) is 2475 bp with five exons and encodes a protein of 597 amino acids, whereas the alternative (202) isoform is 1447 bp but has no opening reading frame and does not encode a protein. Nfe2l2-202 gradually increased in inclusion (at 40CR compared to 12AL) in the eWAT, liver, and stomach at the expense of canonical Nfe2l2-201, although the functional consequences of this switch are unclear. It should be pointed out that both Gna13 [49] and Nfe2l2 [50] have been linked to mitochondrial function, which is an emerging target of AS in both aging and CR.

Two of the other cross-tissue AS loci are involved in the regulation of endosomal sorting and extracellular vesicle biogenesis. Pdcd6ip plays an important role in apoptosis largely as an accessory protein of the endosomal sorting complexes required for transport (ESCRT) machinery [51]. ESCRT is responsible for the formation of endosomal multivesicular bodies (MVBs) and the production of extracellular vesicles (EVs), with EV release being positively regulated by Arrdc4 [52]. Arrdc4 has two protein-coding isoforms annotated in GRCm38, the canonical (201) isoform being 3905 bp with 8 exons encoding a 415-amino acid protein, and an alternative (202) isoform being 1129 bp with 5 exons encoding a smaller 1129-amino acid protein. Arrdc4-201 increased in inclusion (at 40CR compared to 12AL) following CR in the eWAT, hypothalamus and muscle, and decreased inclusion in the liver. Pdcd6ip has six isoforms annotated in GRCm38, although not all were expressed in these data. The canonical (201) isoform is 5951 bp with 18 exons encoding a protein of 869 amino acids, and the alternative (202) isoform is 3223 bp with 18 exons encoding a slightly extended 874-amino acid protein. Pdcd6ip-201 increased in inclusion in the eWAT, liver, muscle, and stomach, with Pdcd6ip-202 following the same pattern in the eWAT and muscle but the opposite in the liver and stomach. The functional consequences of such AS at loci regulating EV biology may suggest that AS-mediated control of circulating EVs, which is increasingly recognized as a factor in senescence and aging [53], is a mediator of the life and health span benefits of CR.

### Limitations

This study has a number of limitations. The most notable are the short sequencing read lengths (50 bp–75 bp) and the shallow library depths (Supplementary Fig. S8). These limitations partly result from some of these data being generated many years ago. Such outdated data significantly reduce the capability to detect and distinguish the expression of transcript isoforms, which itself reflects a limitation of existing sequence mapping algorithms [54]. Moreover, sequencing technology, read length, and library depth differ between some tissues. This reduces the meaningfulness of cross-tissue comparisons, except those concerning within-tissue measurements (such as consistency across CR levels). There are also several limitations in the experimental design. One is that each experimental condition (AL/CR groups) had a relatively small sample size (*n *= 4–8), reducing the overall power. Another is that all animals were male. A third limitation is that the CR intervention only lasted 3 months (short-term). Future studies should attempt to replicate and extend the preliminary results presented here using consistently larger sample sizes and sequencing protocols better suited for isoform-level analyses, such as long-read sequencing. This would provide a more comprehensive assay of isoform expression and diversity that could include unannotated splicing events and be more comparable across tissues. Only a single method was used to assay isoform-level AS. An alternative method based on event-level AS would provide additional qualitative insight into different types of AS changes following CR and their possible regulatory mechanisms. However, event-level AS classification was beyond the scope of the study, which instead provides an overview of global splicing alterations. Including female mice would enable the identification and study of important sex-dependent responses to CR and help prioritize responses most likely causally involved in the benefits of CR. Comparisons with mice undergoing different durations of CR would answer questions about the additive nature of the AS response to CR and allow this response to be associated with the dose-dependent benefits of CR. Accordingly, the results presented here provide a broad but low-resolution characterization of AS in response to CR, and the interpretation of the results depends largely on existing genomic and functional annotations, which did not always appear to be accurate for SRs. Finally, it should be emphasized that all results are purely descriptive and have not been functionally validated or mechanistically explored. We have, however, clearly highlighted the most promising SRs and their predicted roles, providing a strong foundation for future mechanistic and functional studies. Future work should attempt to investigate whether and how specific AS changes following CR identified here are causally involved in age-associated physiological changes relevant to the health and lifespan benefits of CR.

### Conclusion

This study demonstrates that short-term CR evokes a largely tissue-specific but functionally integrated AS program in male mice that is largely independent of expression changes and targets numerous hallmarks of aging, particularly RNA homeostasis, protein homeostasis, and cell death. We identify numerous targets for future mechanistic work investigating the functions of age-associated isoform changes in animal aging and their reversal by CR.

## Materials and methods

### Animal work and sample preparation

Live animal protocols were reviewed and approved by the University of Aberdeen Ethics Approval Committee and implemented under a Home Office-issued license compliant with the Animals (Scientific Procedures) Act 1986. The live animal experiments are fully described elsewhere [27]. Briefly, for 3 months, 48 male C57BL/6J mice (20 weeks old) were fed 10% (*n *= 8), 20% (*n *= 8), 30% (*n *= 7), or 40% (*n *= 9) CR diets, or permitted *ad libitum* (AL) feeding for either 12 h (*n *= 8) or 24 h (*n *= 8) a day (12AL and 24AL, respectively). C57BL/6 mice were used because their lifespan is demonstrably extended by CR [55], and CR was started at 20 weeks old (equivalent to early human adulthood) to avoid confounding effects of altered development [56]. Only male mice were used due to cost limitations. The diet was a semi-purified open-source diet D12450B (Research Diets Ltd), which was carbohydrate-rich (70%) with 20% protein and 10% fat (by energy). CR was individually applied using the pre-experiment (measured over 14 days) baseline daily food intake (e.g. 10CR equals 90% of baseline intake). Food was provided once daily before lights-off (1830 h). For 12AL mice, feeding was restricted to the 12 h of darkness. All mice were housed individually to allow standardization of food intake. The 12AL control group was included to control for confounding effects of rapid food consumption in CR mice, which typically ingest their entire daily ration within hours [57]. Because of this, when animals were culled at the end of the study (1400–1800 h), CR mice may be in a (approximately 10–16 h) fasted state, whereas 24AL mice may have just eaten. This means that the differences between the CR and 24AL mice could in theory reflect a “time since last meal effect”. Limiting feeding of 12AL mice to 12-h dark period only means that they were in a similarly fasted state when culled, thus eliminating confounding effects on biological pathways under circadian control. Mice were culled at about 8 months old using lethal doses of CO_2_ roughly 4 h before their next meal was due (1400–1800 h). Organs were then dissected, weighed, and frozen in liquid nitrogen before being stored at −80°C.

### RNA extraction, library preparation, and mRNA sequencing

Details of RNA extraction and sequencing of the existing RNA-seq datasets (the eWAT [23], liver [24], hypothalamus [25], and stomach [26]) from these mice were documented previously. Sequencing of the novel datasets (the gastrocnemius muscle and testes) was conducted in the same way. Briefly, RNA was isolated by homogenizing tissues using the Tri-Reagent (TRIzol) (Sigma Aldrich, UK) method, following supplier’s instructions. Samples were then denatured at 65 °C before RNA was quantified using an Agilent RNA 6000 Nano Kit.

RNA samples were sent to the Beijing Genomic Institute (BGI, Hong Kong) for RNA-seq. For library preparation, mRNA was first enriched using oligo(dT) magnetic beads and then fragmented into short sequences by adding a fragmentation buffer. Random hexa­mer primers were added to allow the polymerization of the first strand of complementary DNA (cDNA), and the second strand was then polymerized by adding RNase, deoxynucleotide triphosphates (dNTPs), and DNA polymerase. Resulting double-stranded DNA was purified with a QiaQuick polymerase chain reaction (PCR) extraction kit and washed with elution buffer. The purified DNA was then used for end repair and addition of single dATPs. Sequencing adap­ters were ligated to sequences which were then purified by gel electrophoresis and further enriched by PCR. Both Agilent Bioanalyzer and ABI StepOnePlus RT-PCR System were then used to assess the quantity and quality of the prepared sequencing reads. Using primers and sequencing barcodes developed by BGI, and following their in-house protocols, we sequenced the prepared cDNA libraries on an Illumina HiSeq 2000 (to produce 50-bp single-end reads for the eWAT, liver, and hypothalamus) and a BGISEQ-500 sequencer (to produce 150-bp paired-end reads for the testes, stomach, and muscle).

### Data quality and transcript quantification

The quality of the raw Illumina sequencing reads was assessed using FastQC-0.11.8 [58] and MultiQC [59], and reads from tissues with overall adapter sequence contamination > 1 were trimmed using the Cutadapt [60] module of TrimGalore-0.6.4 [61].

Transcript-level quantification was run with Salmon-1.2.1 [62] in quasi-alignment mode, specifying the—seqBias,—gcBias, and—posBias bias-correction parameters, and automatic (-A) sequencing library type detection. Isoform-level AS was assayed rather than event-level AS because it can integrate more intuitively with gene expression and is less impacted by library depth. The GENCODE M25 annotation for the GRCm38.p6 mouse genome was chosen as the reference transcriptome and indexed with Salmon, using a quasi-kmer of 19 bp for the eWAT, liver, and hypothalamus, and 32 bp for the muscle, testes, and stomach, and enabling the —keepDuplicates and —gencode parameters. No samples were removed due to too few reads or a low mapping rate.

### DE analyses

3D RNA-seq was used for data pre-processing and DE analysis [63, 64]. Transcript abundance data were imported using TXimport [65] with the -lengthScaledTPM option specified. Significantly expressed transcript isoforms were those with ≥ 1 count per million (CPM) in ≥ 4 samples of a single tissue, and a significantly expressed gene was one that had ≥ 1 significantly expressed transcript isoform(s). Data for significantly expressed genes and transcripts were then normalized by the trimmed mean of M values method.

The voomWithQualityWeights pipeline of the limma R package [66, 67] was used to identify DEGs and transcript isoforms following short-term graded CR. The change in expression of genes and transcripts was calculated by comparing expression levels in each CR group with those in the 12AL control group. DE results were considered statistically significant if Benjamini-Hochberg-adjusted *P* value (FDR) was < 0.05 and log_2_(FC) was > 0.5.

An *a priori* list of 425 mouse SRs and spliceosome components was assembled to investigate the role of CR in changing the expression of genes regulating AS. The list was constructed by downloading the results of any Gene Ontology (GO)-direct categories containing the word “splicing” and “spliceosomal complex” (GO: 0005681) and filtering by Organism: Mus musculus on the AmiGO free online web application (amigo2.berkeleybop.org/cgi-bin/amigo2/amigo; accessed 16/02/2021).

### DAS analysis

AS was investigated using two different, but complementary full-isoform-level approaches included in the 3D RNA-seq pipeline [63, 64]. Firstly, percentage splice-in (PSI; Ψ) is an isoform-level measurement of relative expression, defined as the proportion of transcripts per million (TPM) expression for a given isoform relative to all other isoforms of the same gene. Accordingly, if all of the expression from a gene comes from a single isoform, this isoform will have a Ψ of 1.0, whereas if the gene has two isoforms with equal expression, both isoforms will have Ψ = 0.5. Isoforms were considered to show significant DTU if ΔΨ  >  5% and Benjamini-Hochberg adjusted *P*-value (FDR) < 0.05, as determined by a modified *t*-test of Ψ between conditions. DAS genes were those where max|ΔΨ| (at least one of its isoforms has ΔΨ) > 5% and the overall proportions of a gene’s isoforms changed significantly across conditions with FDR < 0.05, as calculated by combining, using the Simes method, the individual modified *t*-test *P*-values from comparing each isoform’s log_2_(FC) across conditions against the overall gene log_2_(FC) across conditions.

### GO and pathway enrichment analyses

Both GO and pathway enrichment analyses were performed using the MetaScape online web tool (metascape.org) [68], with multiple gene lists as input and *M. musculus* selected as the organism. The custom enrichment analysis pipeline was run selecting an adjusted *P*-value threshold of < 0.05 and including all possible functional databases. Enrichment map data were further manipulated for visualization in Cytoscape [69]: nodes were sub-set by gene counts per tissue, scaled in size by geneHitsinGOlistandQuery, and colored by tissue. For within-tissue enrichment maps, nodes were colored by CR level: darker colors indicating higher CR levels.

## Supplementary Material

loaf046_Supplementary_Data

## Data Availability

The authors confirm that all the data supporting the findings of this study are available within the supplementary material and corresponding authors.
