# Supplementary material for: A functionally integrated cross-tissue alternative splicing program during short-term calorie restriction
Source: Life Metab. 2026 Feb 12;5(2):loaf046. doi: 10.1093/lifemeta/loaf046 (PMC13110116; doi:10.1093/lifemeta/loaf046)

**Supplementary Figure S1** Enrichment of transcription factor (TF) target genes among differentially expressed genes (DEGs) across six tissues in C57BL/6 mice exposed to graded levels (10%−40%) of short-term (3 months) calorie restriction (CR), compared to mice fed a control diet of *ad libitum* feeding for 12 h (AL12) each day. Heatmap showing the significance (−log_10_(*P* value)) and the change in direction (up- or down-regulated DEGs) of TF target signature enrichment among DEGs in each tissue, at each CR level.


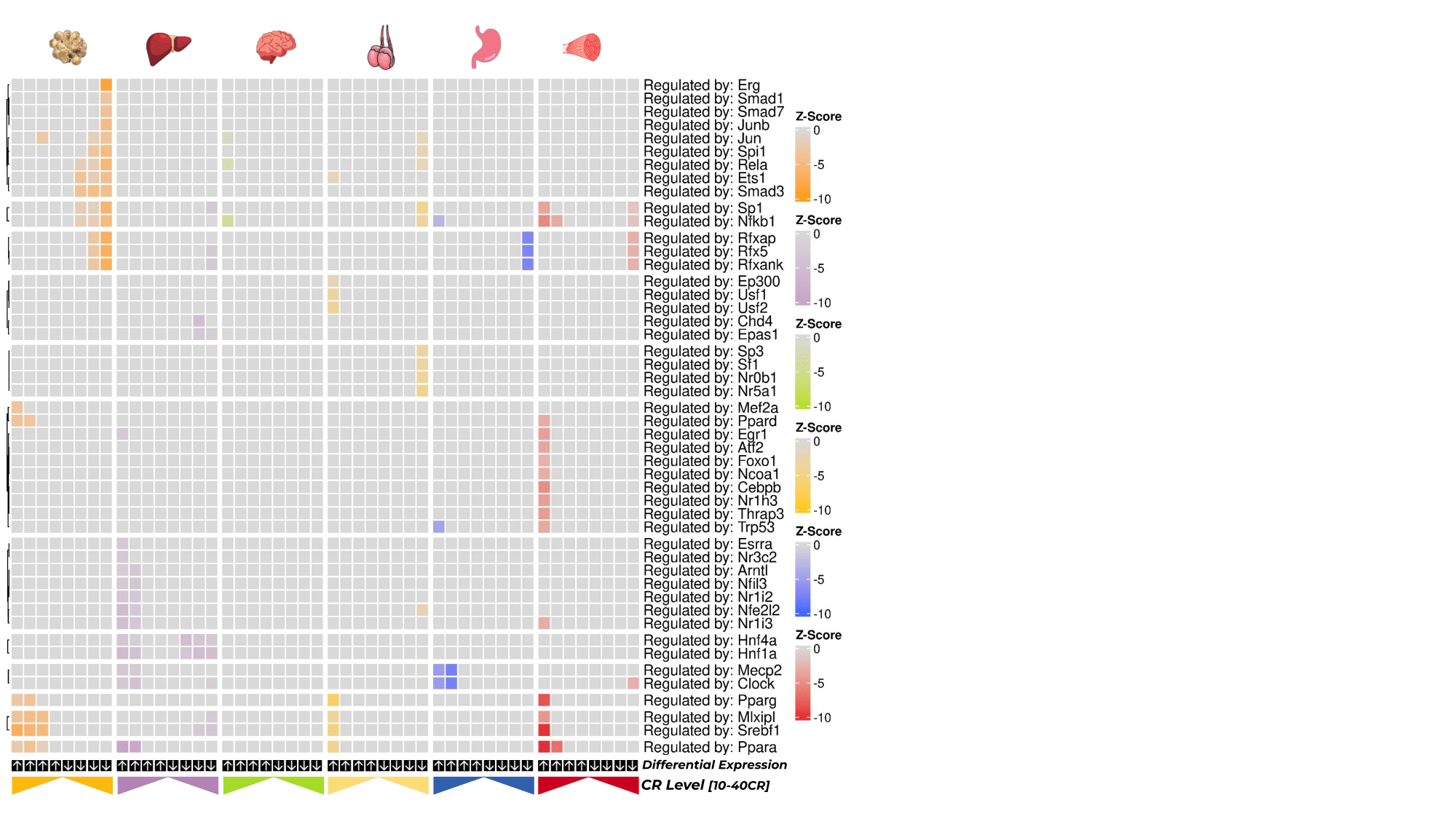


**Supplementary Figure S2** Expression profiles of DEGs in six tissues shared across at least four tissues in C57BL/6 mice exposed to graded levels (10%−40%) of short-term (3 months) CR, compared to mice fed a control diet of AD feeding for 12 h (AL12) each day. Plots showing the gene expression profiles (log2CPM) of a selection of 12 DEGs common across at least four tissues following 40CR compared to AL12, including an indication (end of each row) indicating significance in each tissue (at 40CR; white/black indicates non-significance; tissue-specific colors indicate significance: blue, stomach; green, hypothalamus; yellow, testes; purple, liver; red, gastrocnemius muscle; orange, epididymal white adipose tissue [eWAT]). Statistical significance thresholds were adjusted as *P* value < 0.05 and absolute log2(FC) > 0.5.


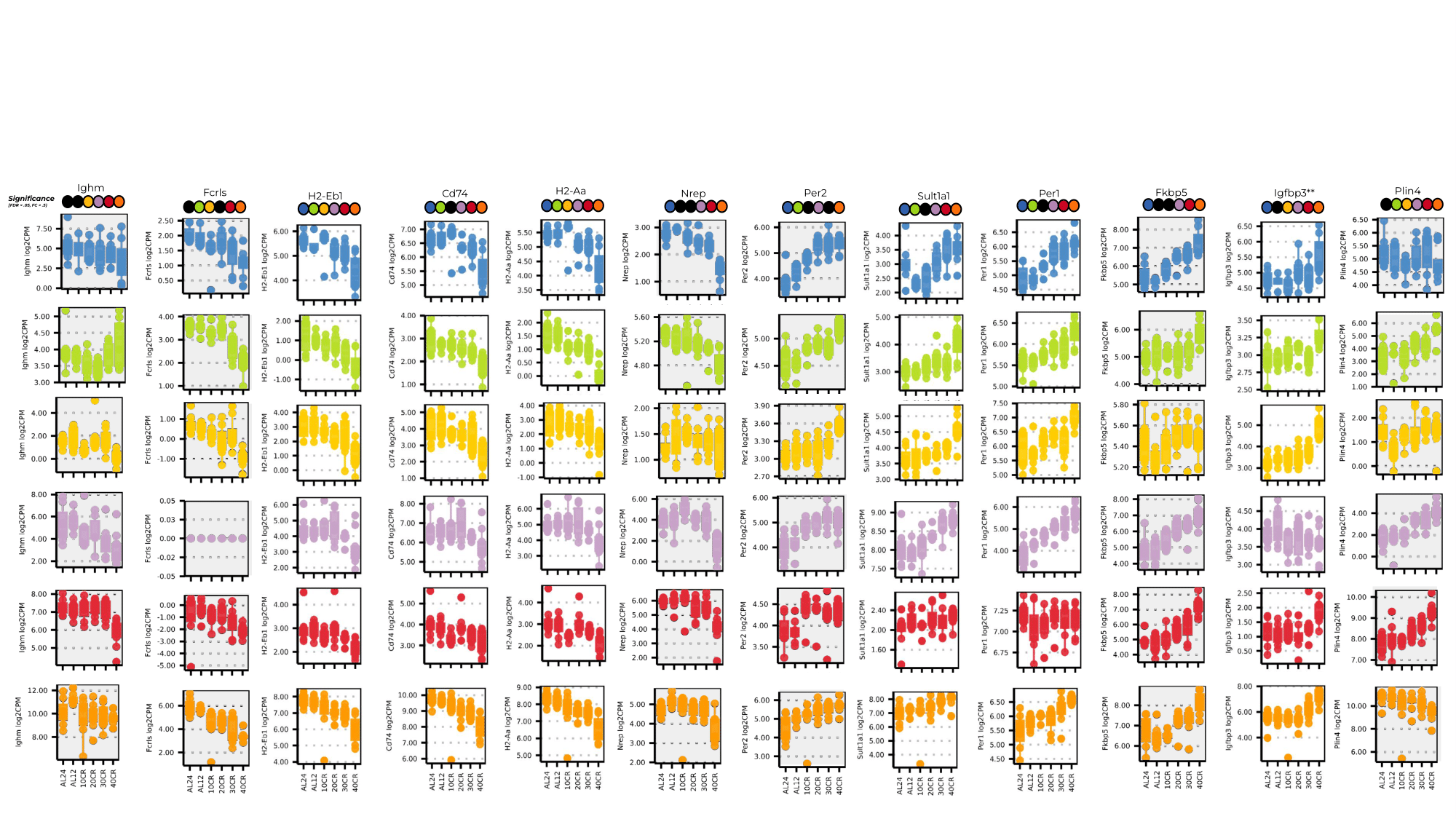


**Supplementary Figure S3** Cross-tissue differential expression of splicing regulators (SRs) in six tissues of male C57BL/6 mice exposed to graded levels (10%−40%) of short-term (3 months) CR, compared to mice fed a control diet of AD feeding for 12 h (AL12) each day. log_2_(CPM) expression profiles of seven differentially expressed SRs common to at least two tissues following short-term CR (at 40CR). Dotted lines indicate genes that are not significantly expressed in a given tissue. Tissue colored are yellow, testes; blue, stomach; green, hypothalamus; purple, liver; orange, eWAT; red, gastrocnemius muscle.


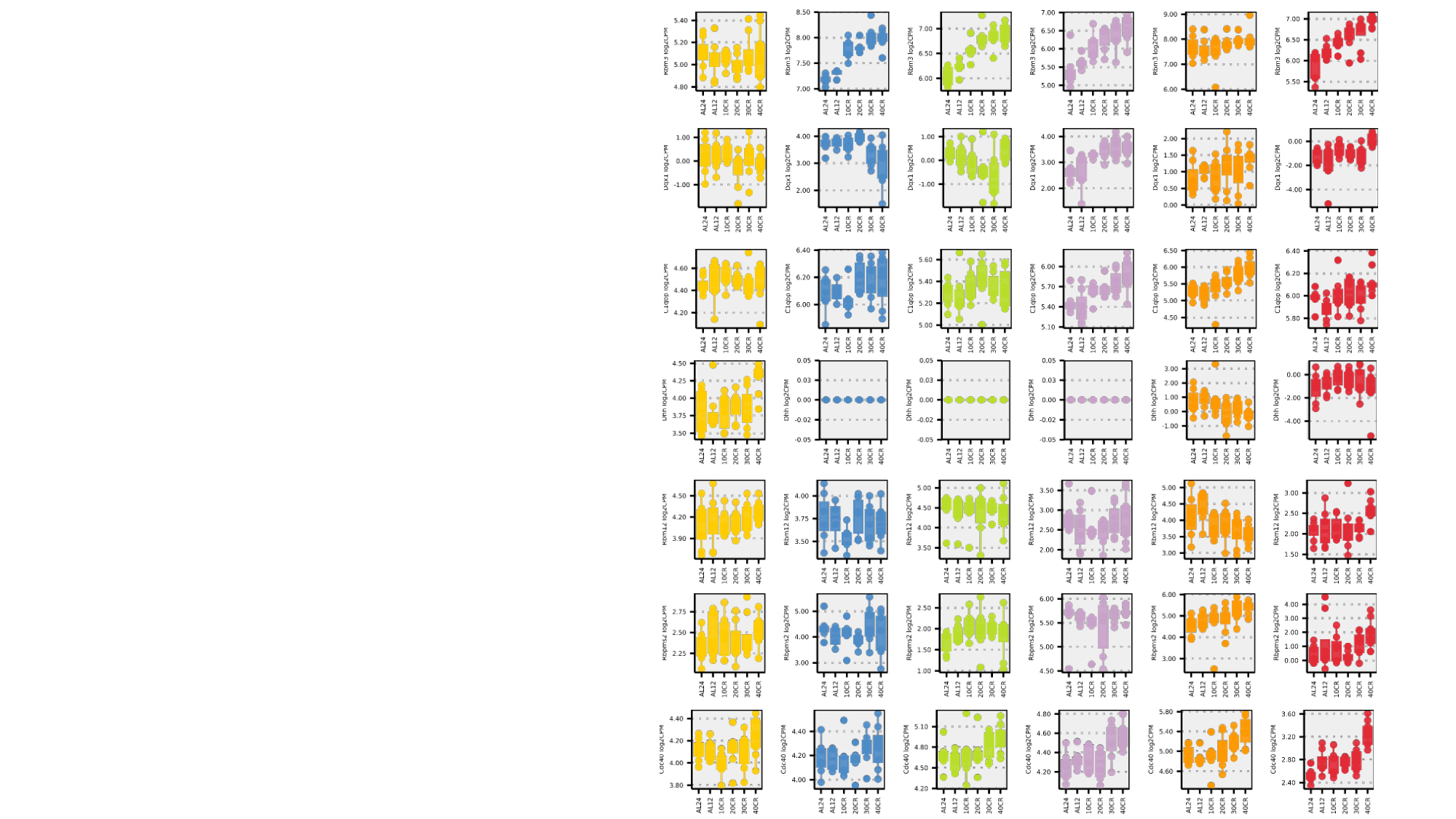


**Supplementary Figure S4** Number of expressed gene transcript isoforms across six tissues in male C57BL/6 mice exposed to graded levels (10%−40%) of short-term (3 months) CR, compared to mice fed a control diet of AD feeding for 12 h (AL12) each day. Grouped bar plot showing counts of the number of transcript isoforms per gene in each tissue after filtering by expression [keeping only transcript isoforms with 1 count per million (CPM) reads in at least four samples].


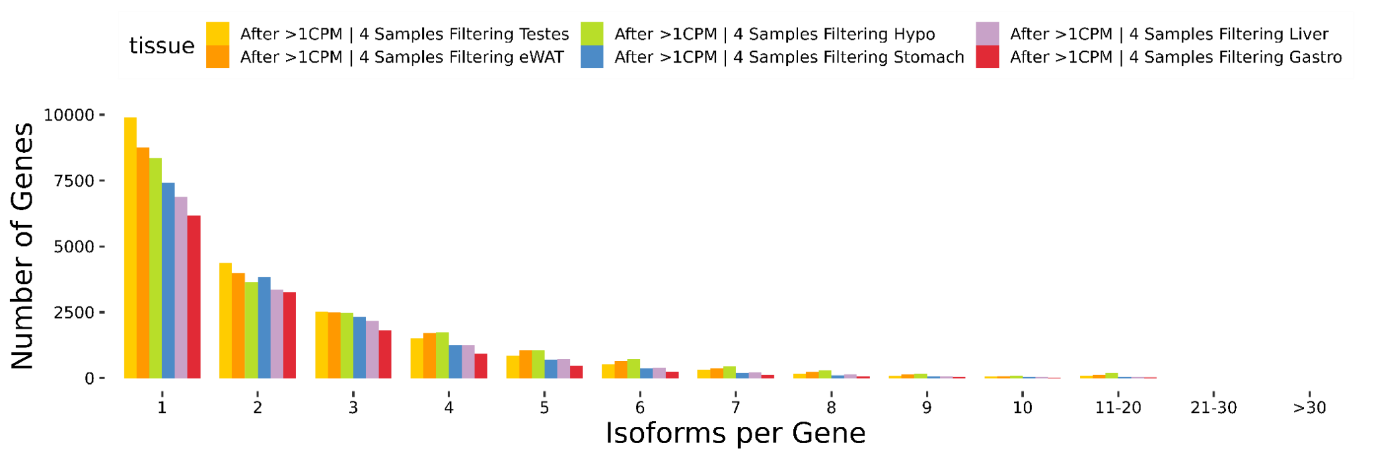


**Supplementary Figure S5** Cross-level alternative splicing (AS) profiles of genes differentially regulated across six tissues in male C57BL/6 mice exposed to graded levels (10%−40%) of short-term (3 months) CR, compared to mice fed a control diet of AD feeding for 12 h (AL12) each day. Heatmaps showing the isoform inclusion profiles (the representational expression of each isoform compared to others of the same gene, denoted as percent splice-in [PSI], scaled by isoform within tissue) of differential transcript usage (DTU) isoforms (compared to AL12) common across 30CR−40CR in each tissue. Representative examples of a small number are additionally shown along with their AS profiles and isoform structures for each tissue. (a) eWAT. (b) Liver. (c) Hypothalamus. (d) Muscle. (e) testes. (f) Stomach.


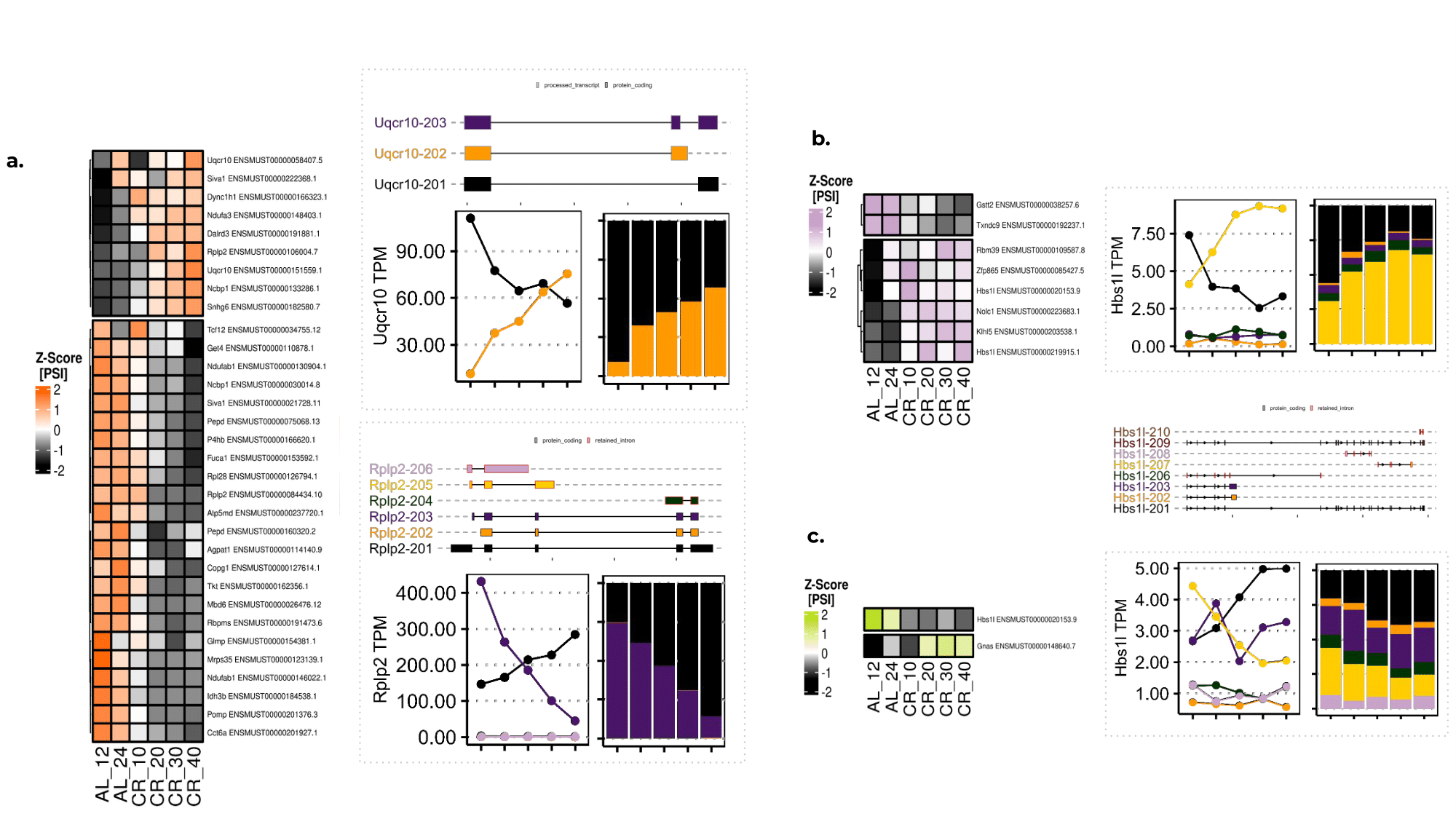

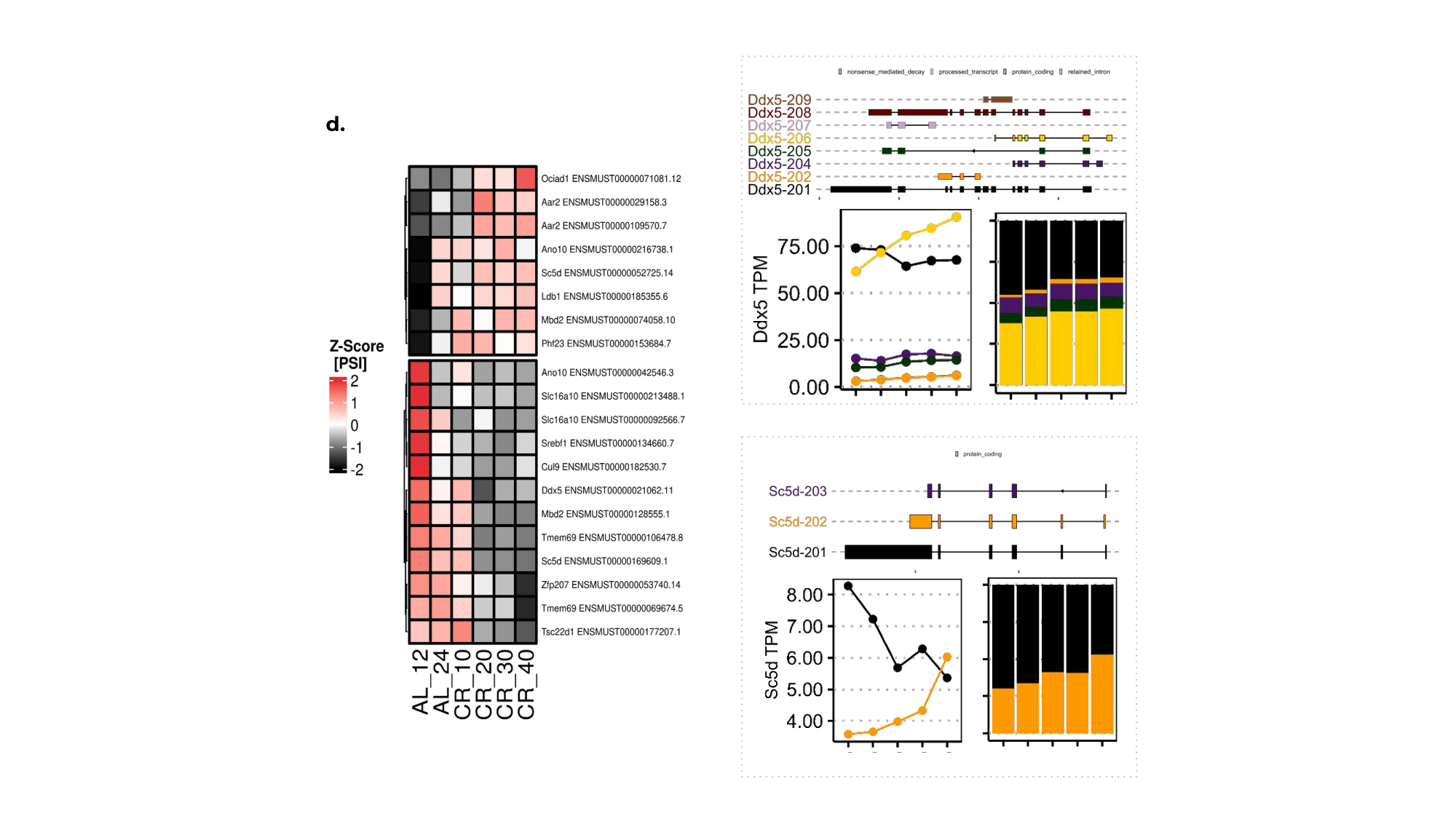

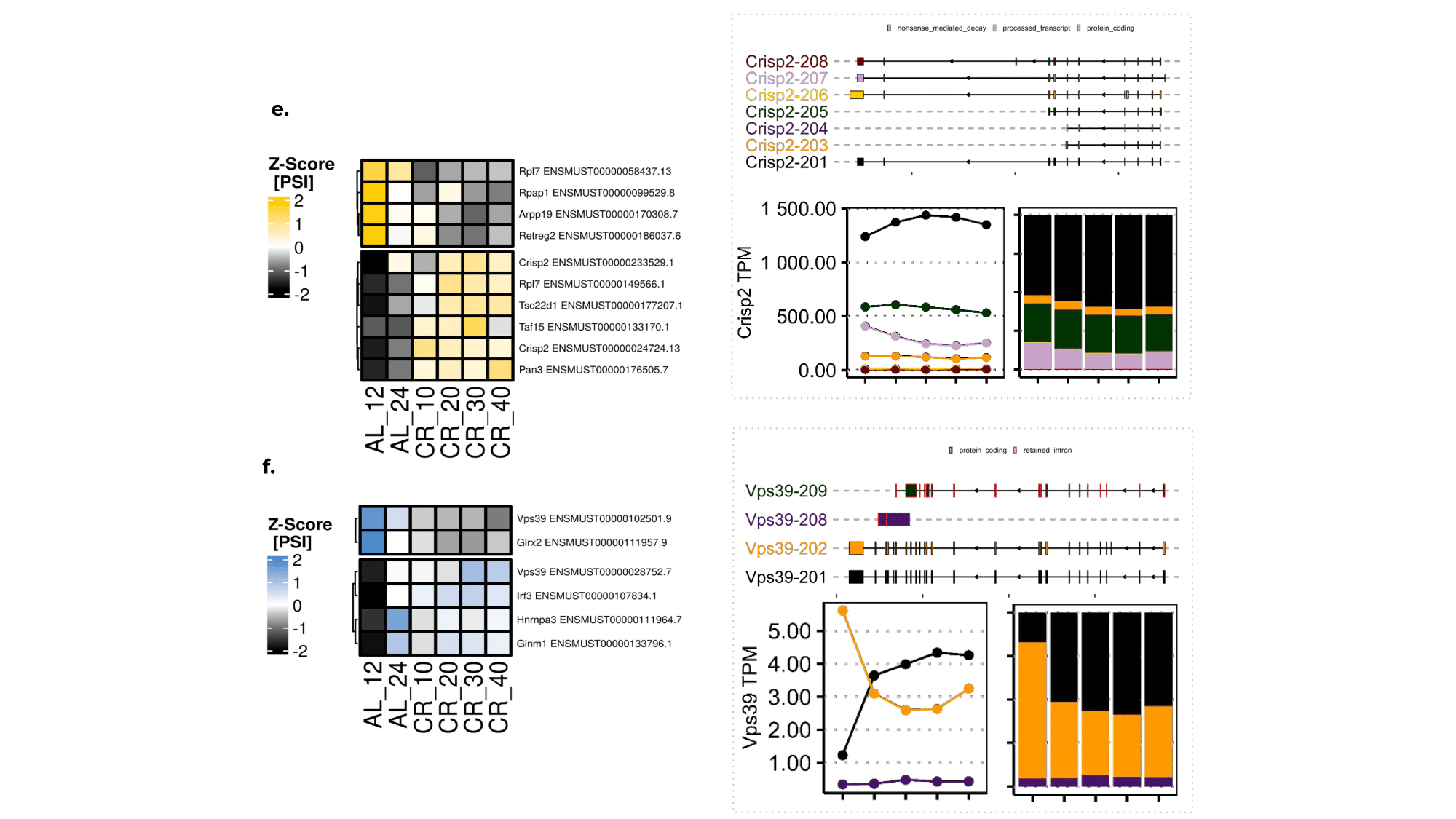


**Supplementary Figure S6** Full results of DTU biotypes of male C57BL/6 mice exposed to graded levels (10%−40%) of short-term (3 months) CR, compared to mice fed a control diet of AD feeding for 12 h (AL12) each day. (a−f) Bar plots showing the number of DTU isoforms at each CR level compared to AL12 in each of the six tested tissues ((a) blue, stomach; (b) orange, eWAT; (c) purple, liver; (d) green, hypothalamus; (e) red, gastrocnemius muscle; (f) yellow, testes) stacked according to their annotated isoform biotypes. NMD, nonsense mediated decay-prone; PT, processed transcript; Pc, protein coding; RI, retained intron; lincRNA, long intervening noncoding RNAs.


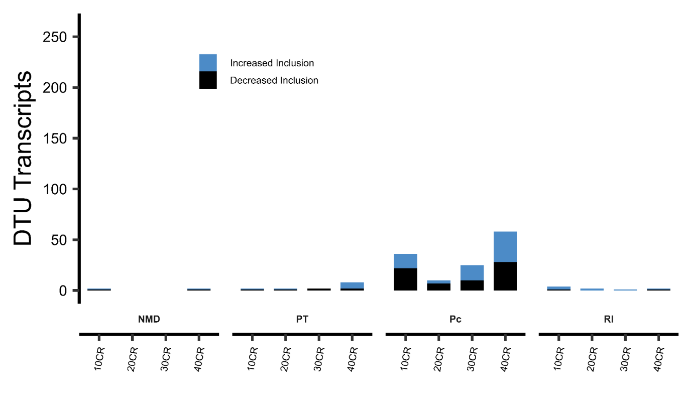

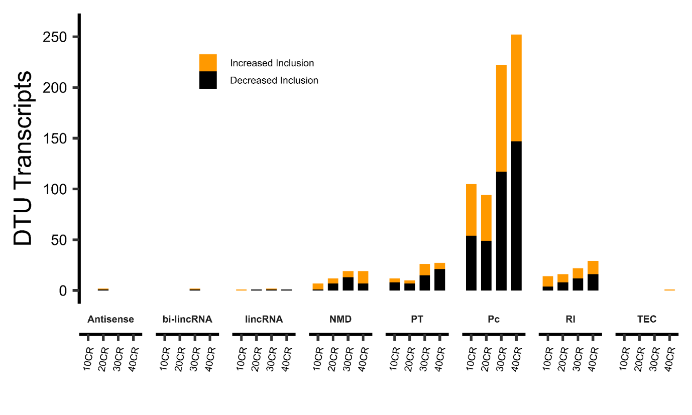

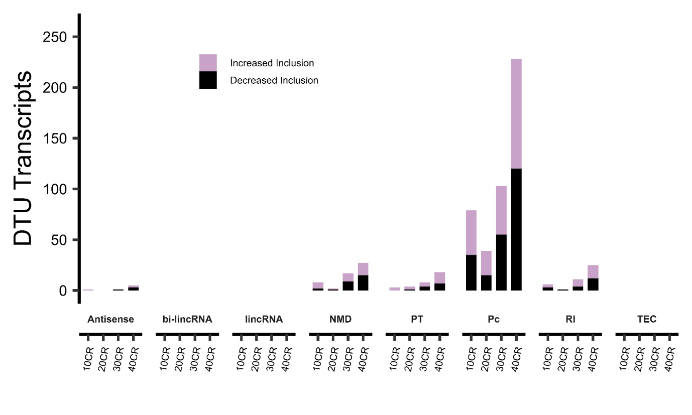

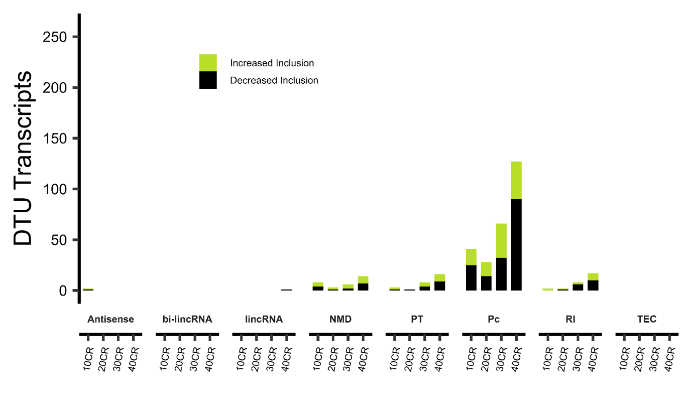

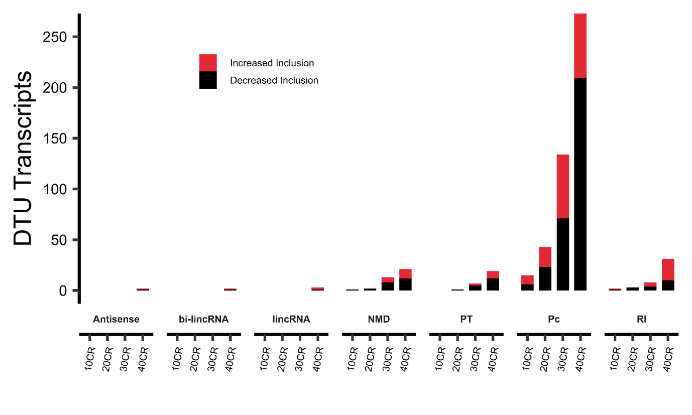

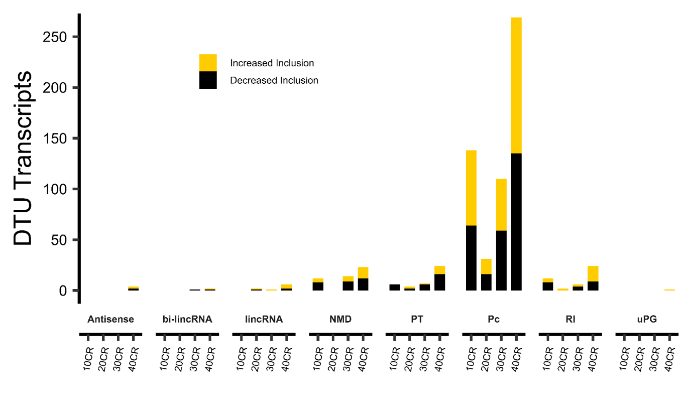


**a)**

**c)**

**e)**

**b)**

**d)**

**f)**

**Supplementary Figure S7** Cross-tissue DTU biotypes of male C57BL/6 mice exposed to graded levels (10%−40%) of short-term (3 months) CR, compared to mice fed a control diet of AD feeding for 12 h (AL12) each day. (a) Upset plot showing the number of DTUs (compared to AL12) common between 40CR across each of the six tissues (blue, stomach; green, hypothalamus; yellow, testes; purple, liver; red, gastrocnemius muscle; orange, eWAT), regardless of their direction of change in each tissue. (b) Combined heatmap showing the scaled (by isoform, within-tissue) AS profiles (the representational expression of each isoform compared to others of the same gene, denoted as percent splice-in [PSI]) of 65 DTUs common across at least two tissues following short-term CR (40CR compared to AL12). Non-significant DTUs are greyed-out to indicate tissue specificity.


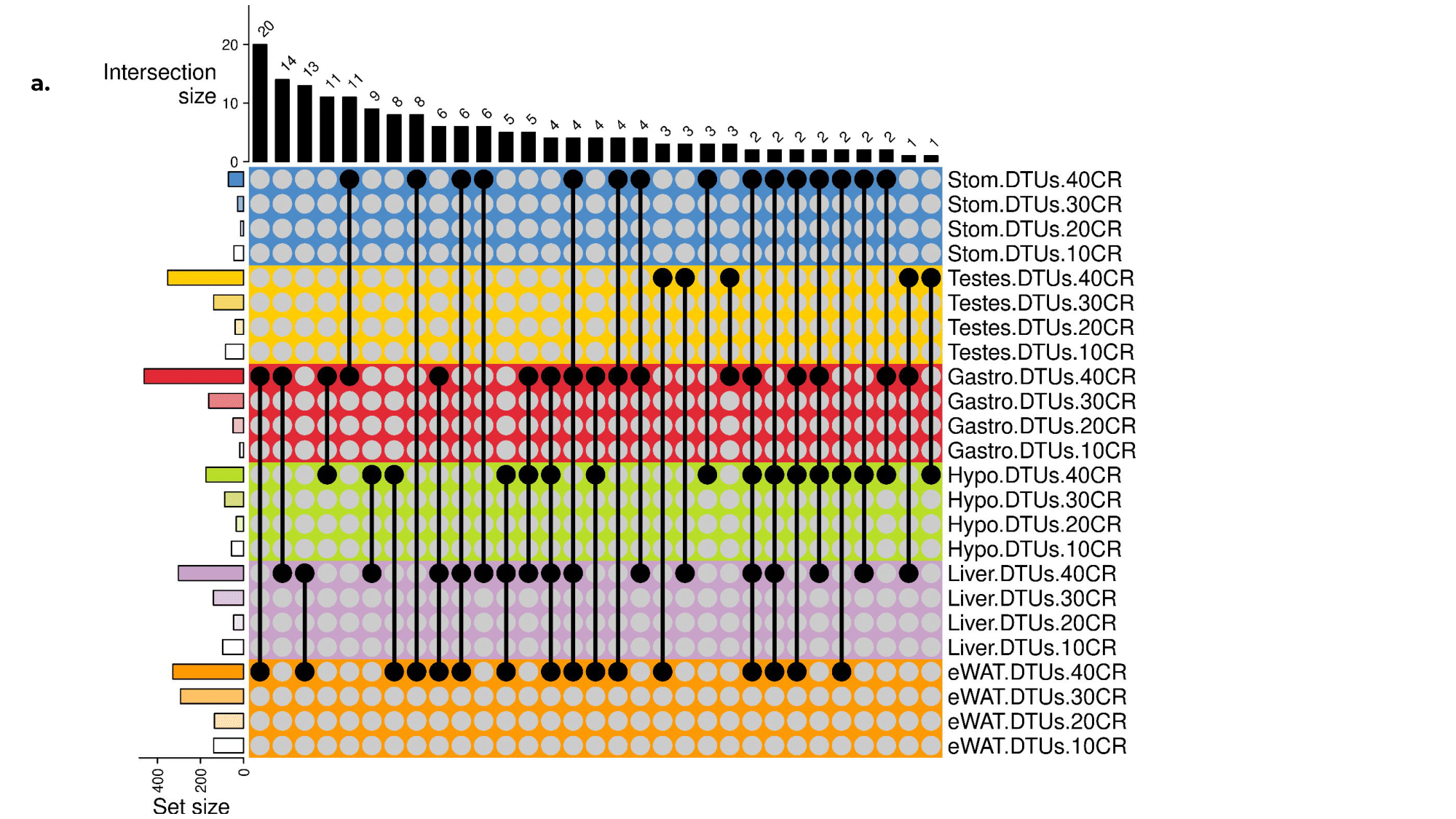


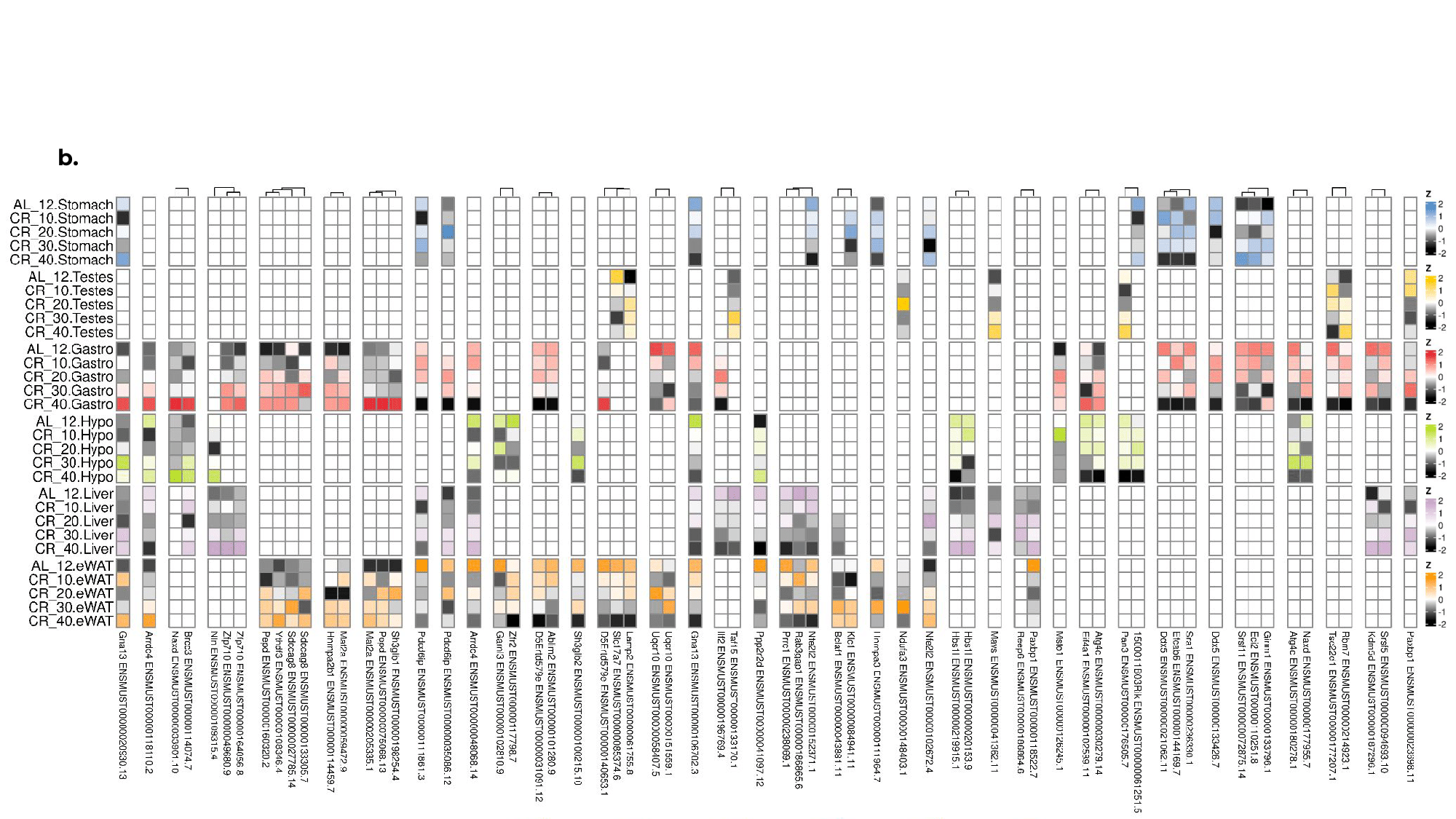


**Supplementary Figure S8** Library depths of RNA sequencing data across six tissues of male C57BL/6 mice exposed to graded levels (10%−40%) of short-term (3 months) CR, compared to mice fed a control diet of AD feeding for 12 h (AL12) each day.


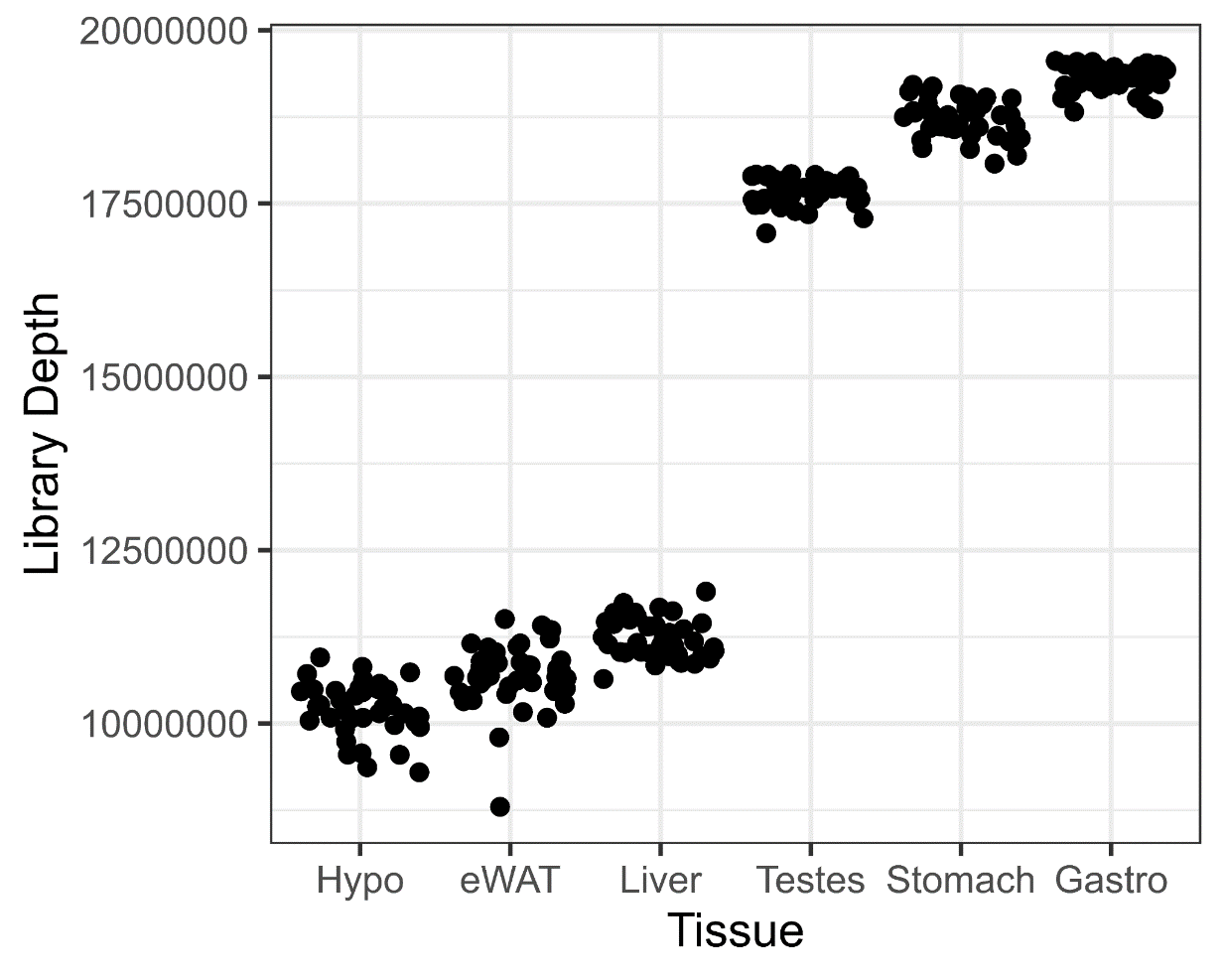

Supplement: loaf046_Supplementary_Data [file loaf046_supplementary_data.zip › Phillips_Supplementary_Figures - tu.docx]
